# The effect of real-time feedback on velocity fluctuations in steady state rowing

**DOI:** 10.1186/2052-1847-7-S1-O5

**Published:** 2015-08-11

**Authors:** Lotte L Lintmeijer, Mathijs J Hofmijster, Knoek J Van Soest, Peter J Beek

**Affiliations:** 1 Vrije Universiteit, Amsterdam, the Netherlands

## Introduction

Rowing performance depends on maximization of mechanical power delivered by the rower(s) and minimization of power losses [1]. Though reductions of power losses may increase the average velocity of a boat, traditional feedback methods lack the accuracy to differentiate between small variations in this power losses. Therefore, we developed a feedback tool to provide real-time feedback about power parameters related to rowing. The current study evaluated the efficacy-with respect to velocity fluctuation power losses-of the real-time feedback on the power loss due to velocity fluctuations compared to the efficacy of traditional feedback.

## Methods

A cross-over design was conducted in which 9 Dutch rowers participated. An algorithm was developed to estimate boat velocity in real-time from combined accelerometer data (sampled at 100Hz) and GPS data (sampled at 10Hz). Power loss due to fluctuations in boat velocity, averaged over each full rowing cycle, was transformed into a single numeric parameter indicating the actual average power loss to boat drag, relative to power loss to average drag. This parameter (one value per full rowing cycle) was fed back visually in real time to single scull rowers, using an android smartphone for both data processing and feedback. In addition, auditory feedback was generated using pitch mapping of the instantaneous power loss due to velocity fluctuations around the mean velocity.

## Results

Multilevel analyses revealed that neither traditional coach feedback nor real-time feedback from the application resulted in a significant decrease of velocity fluctuations due to power loss. No significant differences were found between both conditions. Participants rated the feedback presented by the application positive and would like to row more often with real-time feedback.

## Discussion

Real-time feedback from the application was as effective as coach feedback with respect to reduction of power losses due to velocity fluctuations. No changes of power loss were found in both conditions. Firstly, this may be due to noise with respect to weather conditions which makes it difficult to measure small variations in velocity fluctuation power losses due to feedback. Secondly, rowers may need more time to adjust to the feedback parameter since they are used to train with feedback about velocity. In a next series of studies we will evaluate other relevant power parameters in terms of the power equation for steady state rowing and improve the visibility of the feedback by using Google glasses, ultimately supporting elite rowers to converge to the optimal trade-off between all relevant power terms.
